# Cisplatin resistance-related multi-omics differences and the establishment of machine learning models

**DOI:** 10.1186/s12967-022-03372-0

**Published:** 2022-04-11

**Authors:** Qihai Sui, Zhencong Chen, Zhengyang Hu, Yiwei Huang, Jiaqi Liang, Guoshu Bi, Yunyi Bian, Mengnan Zhao, Cheng Zhan, Zongwu Lin, Qun Wang, Lijie Tan

**Affiliations:** Department of Thoracic Surgery, Zhongshan Hospital, Fudan University, 180 Fenglin Road, Xuhui District, Shanghai, 200032 China

**Keywords:** Cisplatin, Drug resistance, Machine learning, IC50

## Abstract

**Objectives:**

Platinum-based chemotherapies are currently the first-line treatment of non-small cell lung cancer. This study will improve our understanding of the causes of resistance to cisplatin, especially in lung adenocarcinoma (LUAD) and provide a reference for therapeutic decisions in clinical practice.

**Methods:**

Cancer Cell Line Encyclopedia (CCLE), The Cancer Genome Atlas (TCGA) and Zhongshan hospital affiliated to Fudan University (zs-cohort) were used to identify the multi-omics differences related to platinum chemotherapy. Cisplatin-resistant mRNA and miRNA models were constructed by Logistic regression, classification and regression tree and C4.5 decision tree classification algorithm with previous feature selection performed via least absolute shrinkage and selection operator (LASSO). qRT-PCR and western-blotting of A549 and H358 cells, as well as single-cell Seq data of tumor samples were applied to verify the tendency of certain genes.

**Results:**

661 cell lines were divided into three groups according to the IC50 value of cisplatin, and the top 1/3 (220) with a small IC50 value were defined as the sensitive group while the last 1/3 (220) were enrolled in the insensitive group. TP53 was the most common mutation in the insensitive group, in contrast to TTN in the sensitive group. 1348 mRNA, 80 miRNA, and 15 metabolites were differentially expressed between 2 groups (P < 0.05). According to the LASSO penalized logistic modeling, 6 of the 1348 mRNAs, FOXA2, BATF3, SIX1, HOXA1, ZBTB38, IRF5, were selected as the associated features with cisplatin resistance and for the contribution of predictive mRNA model (all of adjusted P-values < 0.001). Three of 6 (BATF3, IRF5, ZBTB38) genes were finally verified in cell level and patients in zs-cohort.

**Conclusions:**

Somatic mutations, mRNA expressions, miRNA expressions, metabolites and methylation were related to the resistance of cisplatin. The models we created could help in the prediction of the reaction and prognosis of patients given platinum-based chemotherapies.

**Supplementary Information:**

The online version contains supplementary material available at 10.1186/s12967-022-03372-0.

## Central message

Through the multi-omics comparison between the cisplatin sensitive and resistant groups, a machine learning model for predicting the effect of cisplatin-containing chemotherapy was established and validated.

## Perspective statement

In this study, we aim to find out the causes of resistance to cisplatin from the genetic, pharmacological, and cellular level as well as the prognosis of patients who have undergone platinum-based chemotherapy, and provide a reference for therapeutic decisions in clinical treatments.

## Central picture legend

Flow diagram of whole design.

## Introduction

Cisplatin was first synthesized by M. Peyrone in 1844 and its chemical structure was first elucidated by Alfred Werner in 1893. However, the compound did not gain sufficient scientific investigations  until the 1960’s, when Rosenberg found that it was capable of inhibiting cell division in Escherichia coli, which increase the possibility of its use in cancer chemotherapy [1, 2].

In 1978, cisplatin became the first FDA-approved platinum compound for cancer treatment [3], and later it became one of the most important anticancer drugs. Nowadays, platinum-based chemotherapy remains an important treatment modality for these patients with advanced NSCLC due to the emergence of resistance to targeted therapies of EGFR, ALK, or ROS mutant tumors [4].

For the mechanism of its pharmacology, generally, they damage DNA, leading to cell cycle arrest and cell death, typically via apoptosis [5, 6]. However, side effects and drug resistance are the two inherent challenges of cisplatin that limit its application and effectiveness [7]. In many common tumor types such as NSCLC, the therapeutic efficacy of platinum-based DNA damaging agents is limited, resulting in only about one-third of patients receive benefits [8, 9]. By now, at least four distinct classes of mechanisms by which cancer cells become resistant to cisplatin-based chemotherapy have been developed and targeting at least two distinct mechanisms might be the most successful strategies for circumventing resistance [10].

In this study, we aim to find out the causes of resistance to cisplatin from the genetic, pharmacological, and cellular level as well as the prognosis of patients who have undergone platinum-based chemotherapy, and provide a reference for therapeutic decisions in clinical treatments.

## Methods

### Data processing

Details of cell lines information were downloaded from Cancer Dependency Map (Depmap, depmap.org) and Cancer Cell Line Encyclopedia (CCLE, https://portals.broadinstitute.org/ccle/data), including IC50 value, cell line source, somatic mutation, mRNA expression, miRNA expression, and metabolite. The information of lung adenocarcinoma patients treated with cisplatin was downloaded from The Cancer Genome Atlas (TCGA, https://gdc.cancer.gov/) (TCGA-LUAD) with their gene expression data. As the CCLE, Depmap, TCGA databases are open to the public under specific guidelines, it confirms that all written informed consents were obtained before data collection.

### Differential analysis

Differential analysis of somatic mutation, RNA, miRNA, and metabolite data between low IC50 and high IC50 groups was performed with R (version 3.6.1). Maftools, the R package, was used to summarize, analyze and visualize the somatic mutation data. RNA, miRNA, and metabolite data were first normalized and standardized by constructing relevant expression matrices using edgfR after removing those without enough sequence fragments in the sample. Differential genes, somatic mutations, miRNAs (P < 0.05, false discovery rate (FDR) < 0.05) were sorted according to logFoldChange values (|logFC|> 1) to identify significantly different expressions. All the differential analyses were presented in a heat map and volcano plots.

### GO, KEGG and GSVA analyses

GO analysis and GSVA analysis were performed to investigate the biological implications of proteins significantly associated with platinum response. R (version 3.6.1) was used for GSVA as well as GO and KEGG pathway enrichment analyses. The significance level was set to 0.05 for the corrected *P*-values. Bar map and dot map were used to visualize the consequences.

### Model contribution

The characteristic of LASSO regression is to consider both Variable Selection and Regularization when fitting a generalized linear model, for applying which, the "Glmnet" (Lasso and Elastic-Net Regularized Generalized Linear Models) R package via penalized maximum likelihood fitness was used, and the mRNAs and miRNAs mostly relative to the resistance to cisplatin were obtained.

Logistic regression, classification and regression tree (CART), and C4.5 decision tree classification algorithm, which use occurrence ratio to determine the category of the dependent variable, was based on the results of LASSO. Finally, we use stepwise regression to select variables, and contribute the predictable model.

### Cell culture and cytotoxic assay

NSCLC A549 and H358 cells were obtained from the American Type Culture Collection (Manassas, VA, USA). Cells were fostered in RPMI-1640 containing 10% fetal bovine serum (FBS) and 100 μg/mL of penicillin–streptomycin with or without DDP (Sigma-Aldrich, Merck KGaA, Darmstadt, Germany) added into the culture medium for incubation in a humid atmosphere containing 5% CO_2_ at 37 °C.

Cell proliferation was evaluated by Cell Counting Kit-8(CCK-8; Dojindo, Kumamoto, Japan). Briefly, 2 × 10^3^ of A549 and H358 cells were plated in 96 well plates. They were incubated with 100 μL RPMI-1640 containing 10% fetal bovine serum (FBS) and 100 μg/mL of penicillin–streptomycin for 24 h, then with or without DDP (Sigma-Aldrich, Merck KGaA, Darmstadt, Germany) for another 24 h at 37 °C. After treatment, cells were incubated in 10% CCK-8 reagent. The OD value was measured after 2 h at 450 nm with a microplate reader from Bio-Rad (Microplate reader 3550-UV).

### RNA interference

siRNAs targeting BATF3, IRF5, ZBTB38, and Silencer Negative Control siRNAs were purchased from Ribobio (sequences provided in Additional file 6: Table S1). We purchased two different siRNAs for each gene to avoid the off-target effects. A549 and H358 cells were seeded in 6-well plates for 24 h prior to transfection with siRNA targeting BATF3, IRF5, ZBTB38, and corresponding non-targeting controls. A total of 150 nM of siRNA was added to each experiment made up of the target siRNA and topped up with the appropriate concentration of non-targeted controls where appropriate. Transfections were carried out in OptiMem medium (Gibco) using Lipofectamine 8000 transfection reagent (Beyotime). 48 h post-transfection, cells were harvested and assayed for RNA and protein expression levels of the target of interest. At the same time, corresponding samples were treated as described in the text.

### RNA preparation and qRT-PCR analysis

To detect the expression of BATF38, IRF5, ZBTB38 in A549 and H358 cell lines, RT-qPCR was carried out on an QuantStudio^®^ 5 real-time PCR system (Applied Biosystems) with proper PCR parameters.

Total RNAs were extracted by TRIzol (TIANGEN, Beijing, China). The first-strand cDNA was synthesized using Hifair® III 1st Strand cDNA Synthesis SuperMix for qPCR (gDNA digester plus) (YEASEN, Tokyo, Japan) according to the manufacturer's instructions. Then Hieff^®^ qPCR SYBR Green Master Mix (Low Rox Plus) (YEASEN) was used with the following PCR parameters, 1 cycle of 30 s at 95 °C, 40 cycles of 5 s at 95 °C and 34 s at 60 °C. β-actin was used as the reference. Primers used in this study are listed in Additional file 7: Table S2.

All the samples were repeated three times.

### Western blot analysis

Proteins of A549 cells and H358 cells after RNA interference were extracted using RIPA (Beyotime, Shanghai, China) with protease and phosphatase inhibitor cocktail (Topscience). Then these proteins were quantified by Enhanced BCA Protein Assay Kit (Beyotime). Proteins were then resolved, separated, and finally transferred into PVDF membranes under the influence of an electric current in a procedure (Merck-Millipore, Burlington, MA, USA). Membranes were blocked, followed by incubation with specific primary antibodies [11].

Finally, we observed the protein bands by Moon chemiluminescence kit (Beyotime). The following antibodies were used: Rabbit anti-BATF3 (NBP2-41296, dilution 1:1,500, Novus Biologicals); Rabbit anti-IRF5 (CY5822, dilution 1:1,500, Abways); rabbit anti-ZBTB38 (21906–1-AP, dilution 1: 1,500, Proteintech), mouse β-ACTIN (1:3,000, AA128, Beyotime), horseradish peroxidase (HRP)-labeled goat anti-rabbit IgG (H + L) (1:3,000, A0208, Beyotime), and HRP-labeled goat anti-mouse IgG (H + L) (1:3,000, A0208, Beyotime).

### Single tumor and immune cells

We used the same methods described in our previous studies to test cisplatin-sensitivity-related genes [12].

This study was approved by the Ethics Committee of Zhongshan Hospital, Fudan University (B2019–137R). Patients had signed the informed consent at hospitalization.

## Results

### Group division and overview of cells’ IC50 value of cisplatin

**Fig. 1:**
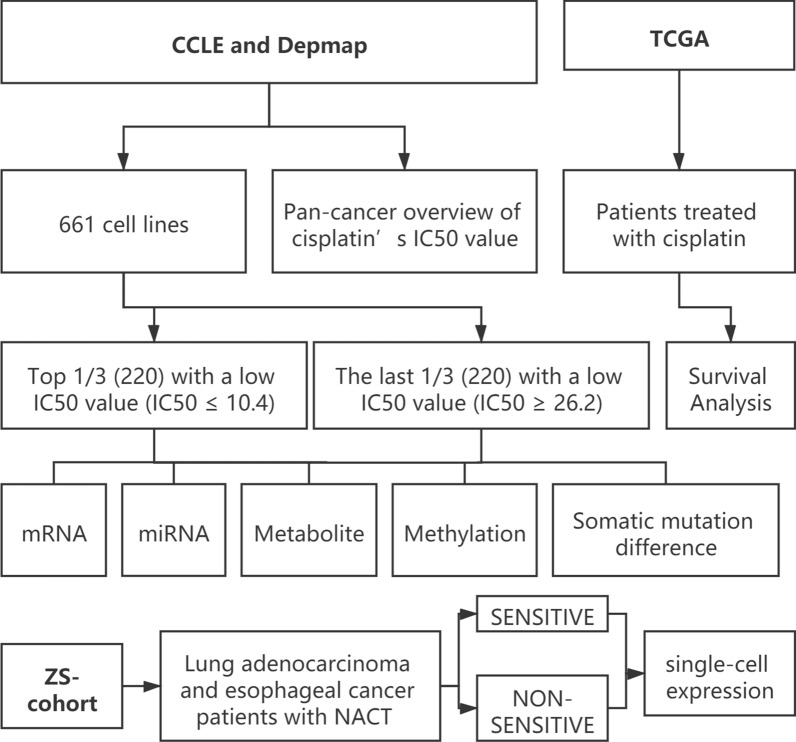
Flow diagram of whole design

661 cell lines were finally enrolled with the complete data of mRNA, microRNA expression, and metabolite. They were divided into 3 groups according to the IC50 value, the half inhibitory concentration, given by the CCLE database, which reflects the sensitivity to chemotherapeutic drugs. The lower the value, the stronger the sensitivity. In this study, the top 1/3 (220) with a low IC50 value (IC50 ≤ 10.4) were defined as the sensitive group, while the last 1/3 (220) with a low IC50 value (IC50 ≥ 26.2) were enrolled in the insensitive group (Fig. 1).

Obviously, the resistance to cisplatin varies among different cancers. In the Depmap database, we successfully classify different tumor cell lines according to their source of cancer, so as to obtain the average IC50 value of cisplatin of related cancers. As shown in Additional file 1: Fig. S1, the average IC50 value of 6 cancers were more than 100, including Lung adenocarcinoma (LUAD), Mesothelioma (MESO), Thyroid carcinoma (THCA), etc., which means patients with these types of cancer may suffer a higher possibility of resistance to cisplatin, one of the most common chemotherapy drugs.

### Somatic mutation difference

**Fig. 2:**
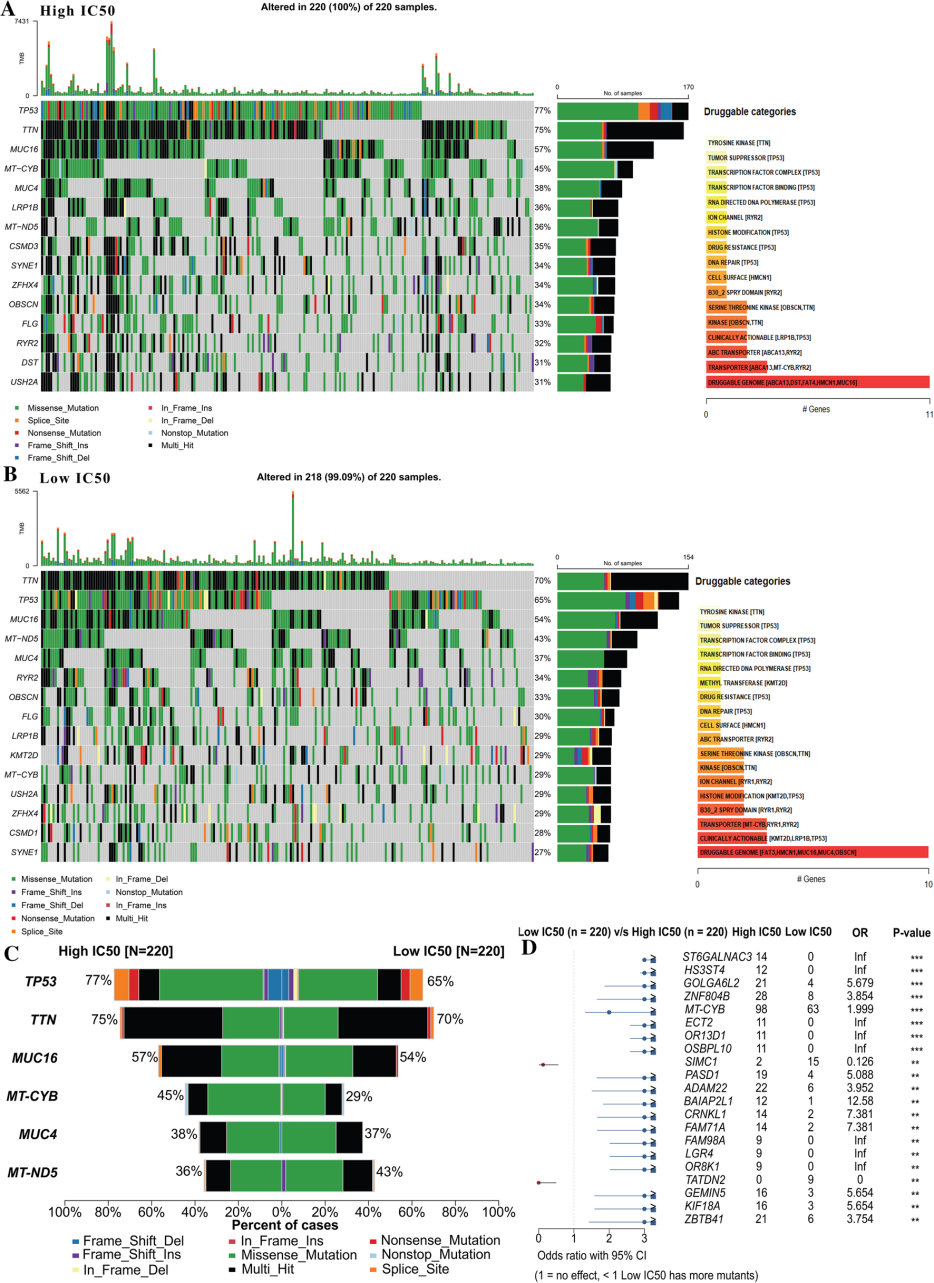
Somatic mutation difference between high and low IC50 groups of cisplatin; **A** refers to the low IC50 group; **B** for the high IC50 group; **C** for the compartments between 2 groups ranked by the rate of difference; **D** top 15 different mutations between 2 groups ranked by P-value

The overall pattern of somatic mutation of the cell line in detail was described in Additional file 2: Fig. S2. After matching the somatic mutation data with the drug sensitivity data, the differences in the characteristics of somatic mutation were investigated between the low IC50 (218 in 220) and high IC 50 (220 in 220) groups. As shown in Fig. 2A and B, although two somatic mutation oncoplots and co-occurring sets of these genes are similar to a certain extent, it is not difficult to find that in the cisplatin-resistant group (high IC50), the mutation of TP53 ranked the first (10%), which is higher than the mutation rate in the cisplatin-sensitive group (low IC50 group). As previously reported by Aditya Bagrodia [13], TP53 mutations are only found in cisplatin-resistant tumors, especially in primary mediastinal non-seminoma. Therefore, we might conclude that TP53 mutation is closely related to cisplatin resistance. In contrast, MUT5 had a higher mutation rate in the low IC50 group. We also checked for drug-gene interactions compiled from Drug Gene Interaction database with "drugInteractions" function. Most drugs are related to TTN and TP53, which is less different between the two groups of cells.

### Differential analysis from different levels gene functional analysis

**Fig. 3:**
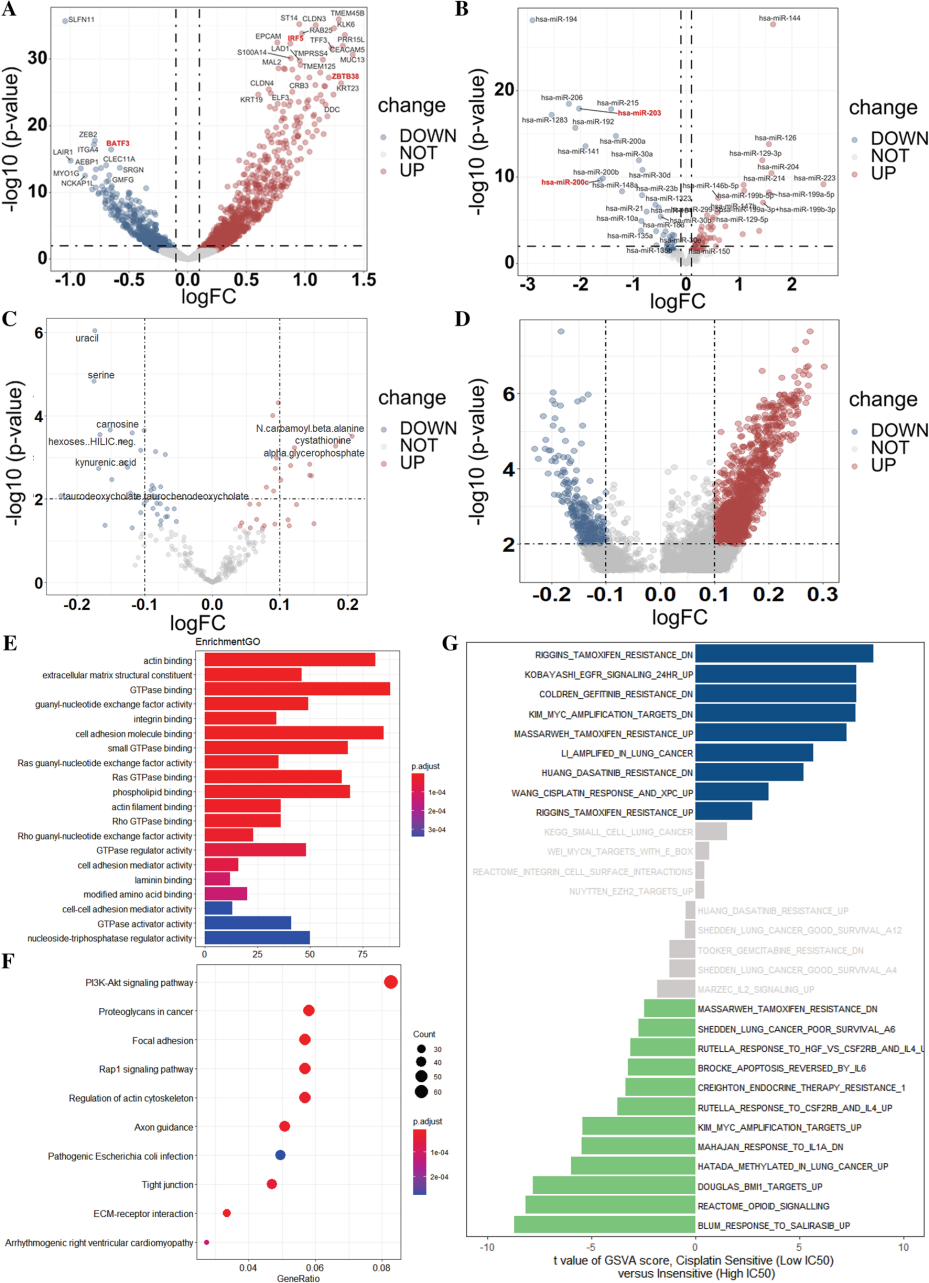
Differential analysis from different levels between cisplatin sensitive and resistant group; **A** for the mRNA level; **B** for the miRNA level; **C** for the metabolism level; **D** for the methylation level; **E**–**G** Pathway analysis of the different coding genes; **E** for GO; **F** for KEGG; **G** for GSVA

To further discover the differences between the 2 groups, differential analysis between the two cell lines was applied from different levels. First, at the mRNA level, through edgfR, we found 1348 mRNA were differentially expressed between two groups (P < 0.01), including 1 downregulating in the cisplatin resistance group (SLFN1) and 35 upregulating (TMEM4SB, CLDN3, KLK6, IRF5, ZBTB38, etc.) with an obvious fold change (|logFC| > 1) (Fig. 3A). Similarly, at the miRNA level, as shown in Fig. 3B, 80 differentially expressed miRNA were discovered (P < 0.01) and 11 downregulating (miR-194, miR-206, miR-215, etc.,), 12 upregulating (miR-144, miR-16, miR-129-3p, etc.,) with an obvious fold change (|logFC| > 1). However, for the metabolizes, only the expression of 15 metabolites, such as urail, serine, carnosine, N-carbamoyl-β-alanine, cystathionine, etc., showed significant differences between the two groups, with no obvious fold change (|logFC| < 1) (Fig. 3C). For the methylation, none of the differential methylation sites with obvious fold change (|logFC| > 1) were closely related to the differentially expressed genes (Fig. 3D).

We firstly performed a Gene Ontology (GO) analysis of the 1348 mRNAs that were found differentially expressed before based on the CCLE data. Gene functional enrichment analysis showed that 20 GO functional groups exhibited significant differences between the low IC50 group and the high IC 50 group, including actin-binding, extracellular matrix structural constituent, GTPase binding, guanyl-nucleotide exchange factor activity, etc. (Fig. 5A). For Kyoto Encyclopedia of Genes and Genomes (KEGG) based on these mRNAs (Fig. 5B), 10 pathways differed between two groups: PI3K-Akt signaling pathway, which had the most significant difference and the highest gene ratio, followed by proteoglycans in cancer, focal adhesion, etc.

Next, to further conclude the difference of pathways and functions, we applied the Gene set variation analysis (GSVA), which takes specific gene sets as a characteristic expression matrix and quantifies the results of gene enrichment (Fig. 5C). In all, we concluded 643 gene pathways, most of which were related to drug resistance, including Riggins tamoxifen resistance, EGFR signaling 24HR, gefitinib resistance, KIM-MIC amplification targets, etc.

### Model construction and evaluation

**Fig. 4:**
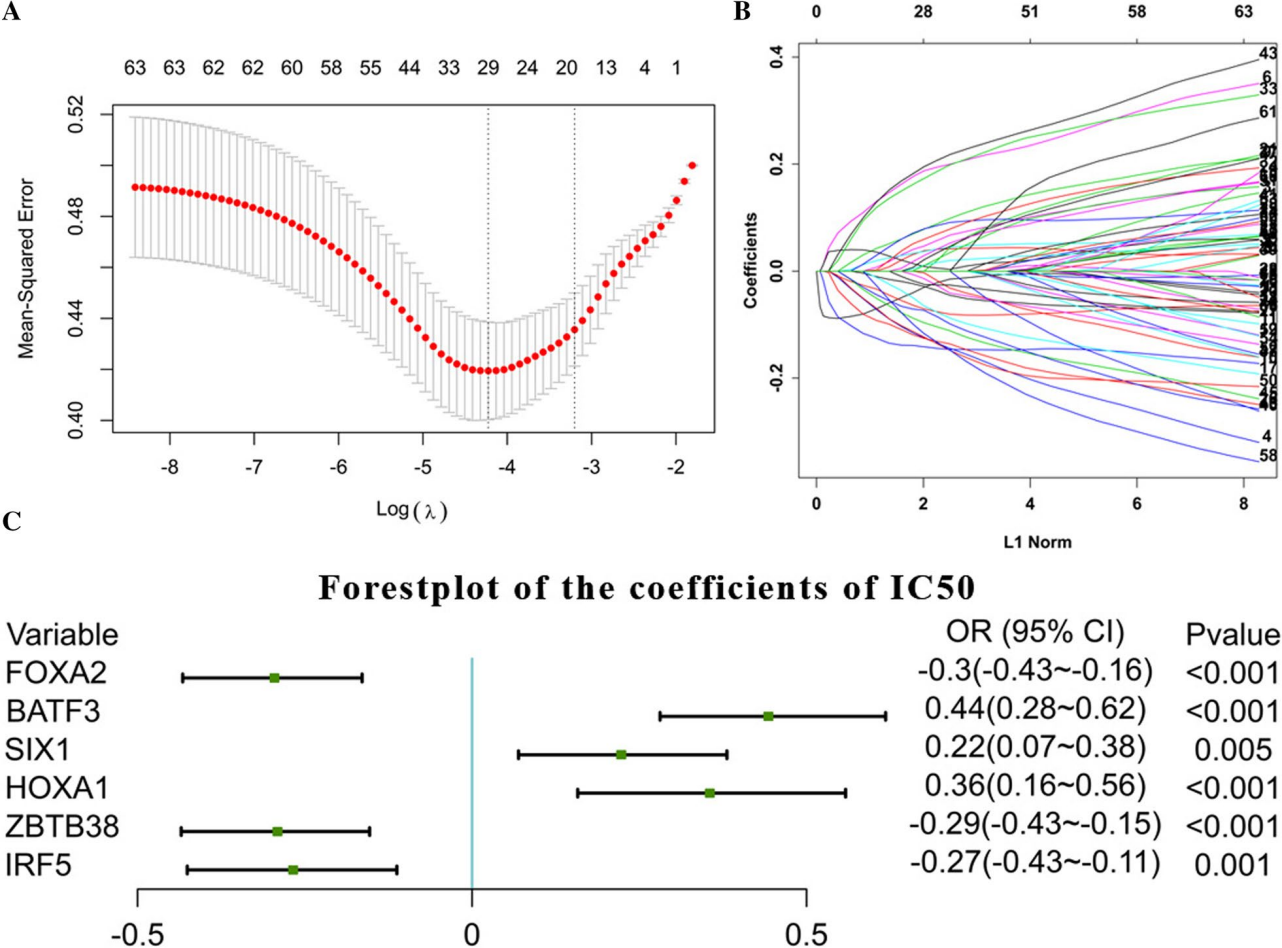
cisplatin-sensitivity related model. **A**, **B**. Establishment of the LASSO model; **C** Coefficient display of logistics regression equation model of cisplatin-sensitivity related genes

Firstly, the glmnet R package was applied with "family = binomial", which is suitable for binary discrete dependent variables to determine whether the DEGs were related to the high or low IC 50. 6 of the 1348 mRNAs, FOXA2, BATF3, SIX1, HOXA1, ZBTB38, IRF5, selected as the associated features with cisplatin resistance (Fig. 4A, B), and then related logistic model was established through glm [14] function (Fig. 4C).

Next, we applied J48 and rpart R package with ""formula = ic50 ~ FOXA2 + BATF3 + SIX1 + HOXA1 + ZBTB38 + IRF5"" and ""family = gaussian"", which is suitable for continuous univariate as we aimed to get the predicting value of IC50 value through this model (Additional file 3: Fig. S3). The step function is used to achieve stepwise regression. From the results, it is found that 3 in 6 variables had passed the significance test (p < 0.05) and became relatively important variables for constructing the model. While each variable in the model passed the significance test, it is necessary to ensure that the entire model is significant. Therefore, the Chi-Squared test was performed on the model. As the variables were added to the model one by one from the first to the last, the model finally passed the significance test, indicating that the model composed (Table 1) of these variables is meaningful and correct.

**Table 1:** Coefficient display of logistics regression equation model for miRNA

Intercept	has-miR-203	has-miR-200c
27.806962754	0.013017718	0.003779486

**Fig. 5:**
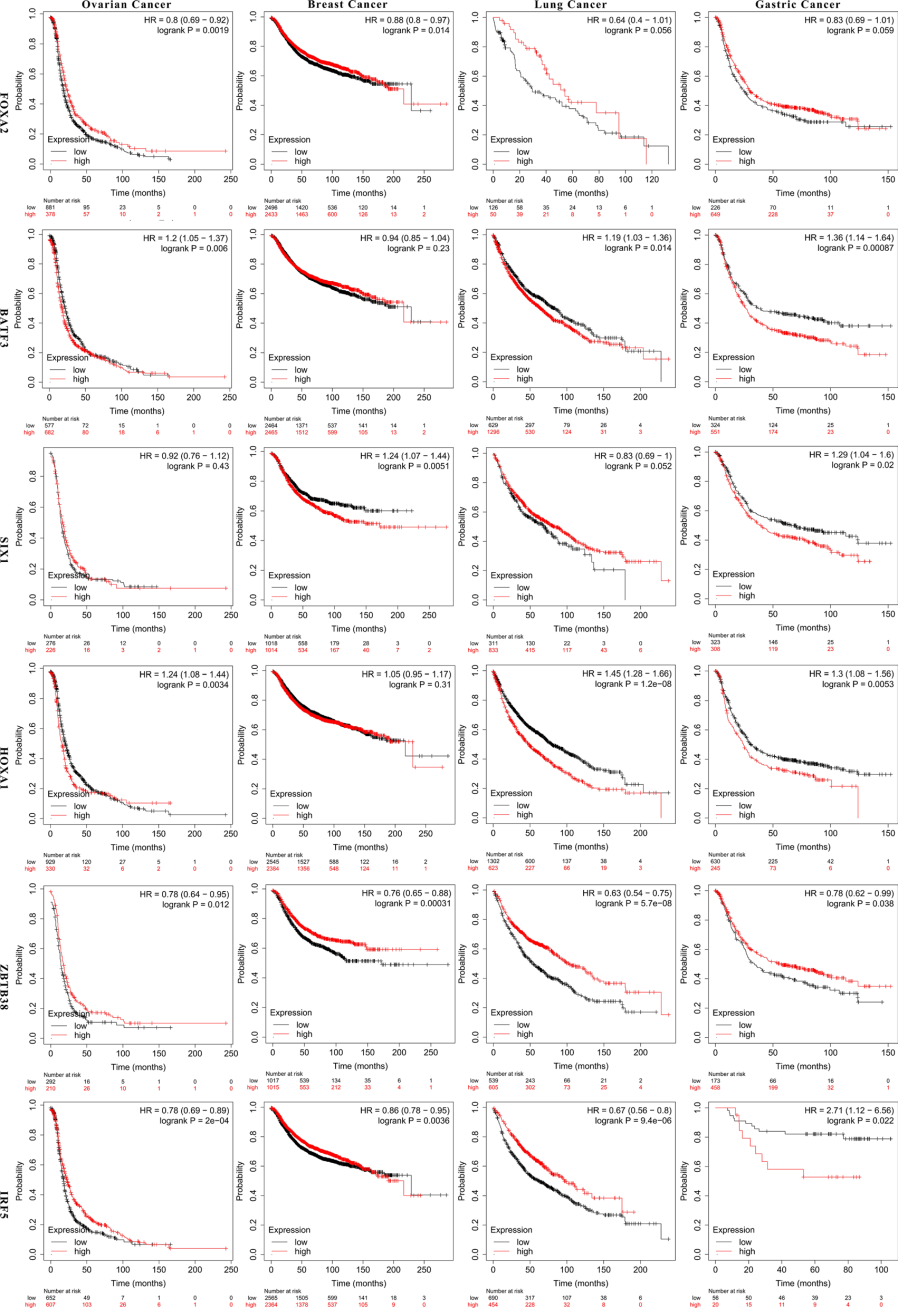
K-M plot showing the differences of survival time between high and low expression of FOXA2, BATF3, SIX1, HOXA1, ZBTB38, IRF5

Finally, we testified whether these genes are not only related to cisplatin resistance, but also have connection with patient survival. In addition, according to the data in the TCGA database, their expression levels were related to the prognosis of different tumor patients, including breast cancer, ovarian cancer, lung cancer and gastric cancer to a certain extent (Fig. 5). Indeed, BATF3, IRF5 and ZBTB38 also contributed in evaluation of the response to chemotherapy of breast and ovarian cancer patients (Additional file 4: Fig. S4).

Similarly, we composed the miRNA predicting model. From the LASSO regression, 4 significant miRNAs were concluded, miR-203, miR-200c, miR-148a, miR-142-5p (Additional file 5: Fig. S5). After the step function, miR-203 and miR-200c left, both of which passed the significance test conducted by Chi-Squared test, indicating that the model composed (Table 1) of these variables is meaningful and correct.

### Expression of BATF3, IRF5, ZBTB38 is associated with the sensitivity of cisplatin in A549 and H358 cells and tumor samples

**Fig. 6:**
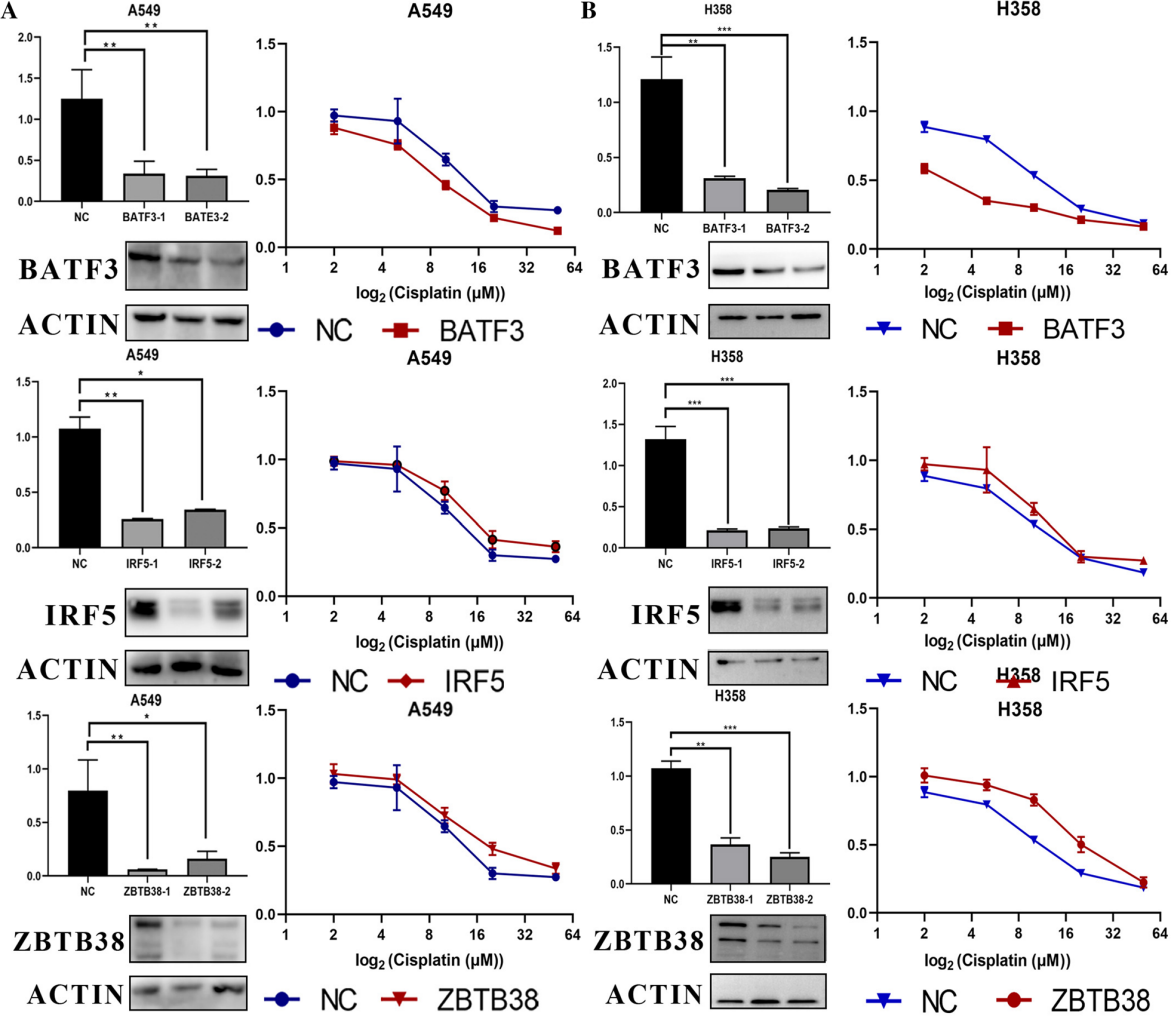
Silencing BATF3 significantly increases sensitivity to cisplatin, while silencing IRF5, ZBTB38 reduces. **A**, **B** qRT-PCR and Western blot showing BATF3, IRF5, ZBTB38 knockdown 3 and 4 days after transfection with BATF3-targeting, IRF5-targeting and ZBTB38-targeting siRNAs with cytotoxicity curves of the lung adenocarcinoma cell line A549 and H358 transfected with nontargeting (ctrl) or BATF3-targeting, IRF5-targeting and ZBTB38-targeting siRNAs and treated for 48 h with cisplatin. **C** Immunohistochemistry for NACT sensitive and insensitive LUAD patients' tumor tissue

To test the causal relationship between BATF3, IRF5, ZBTB38 expression and sensitivity to cisplatin on the cell level, we transfected BATF3-targeting, IRF5-targeting, and ZBTB38-targeting siRNAs separately in human cancer cell lines, A549 and H358 (non-small-cell lung cancer), which on the one hand expresses high BATF3, IRF5, ZBTB38 levels. These two cell lines in the result of previous sequencing analysis, and on the other hand, were constantly used in our lab. All siRNA-mediated silencings proved to be highly efficient and were sustained for 3d or more. IRF5 and ZBTB38-silenced cells exhibited at least a reduced sensitivity to cisplatin compared with mock-silenced cells, while the BATF3-silenced cells exhibited increased sensitivity to cisplatin (P ≤ 0.01, Fig. 6A, B).

**Fig. 7:**
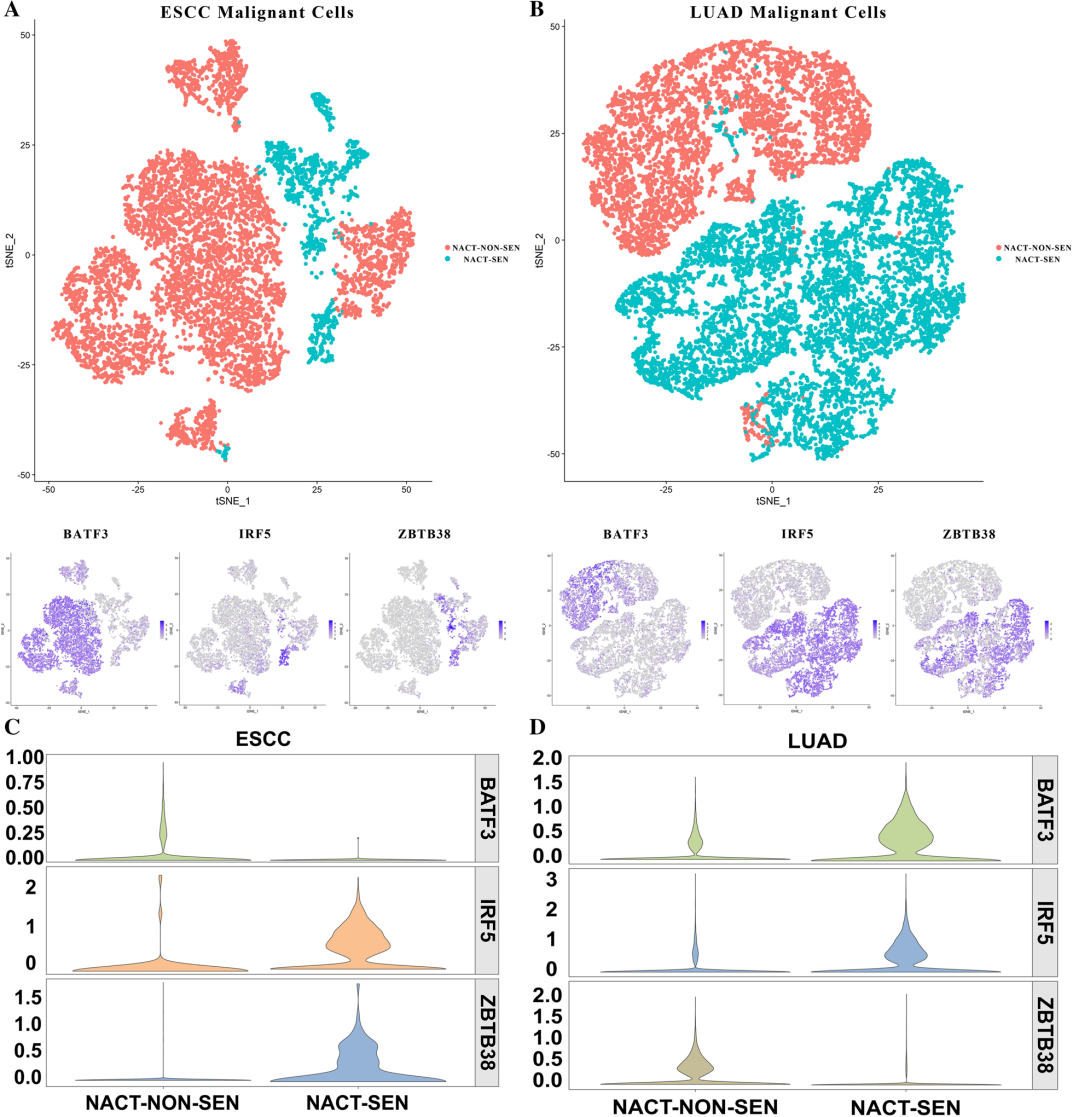
tSNE of tumor cells, BATF3, IRF5, ZBTB38 in NACT-NON-SEN and NACT-SEN groups in ESCC (**A**) and LUAD (**B**). Violin plots showing the difference of expression of the 3 genes between NACT sensitive and insensitive ESCC (**C**) or LUAD (**D**) patients for malignant cells

A total of 6 ESCC (Esophageal Squamous Cell Carcinoma) patients were selected, who accepted complete cisplatin-containing neoadjuvant chemotherapy treatment, including 4 neoadjuvant-chemotherapy insensitive (NACT-NON-SEN) tumor samples and 2 neoadjuvant-chemotherapy sensitive (NACT-SEN, complete response [CRs]) tumor samples (Fig. 7A). RECIST standard was used to evaluate the effectivity of NACT effect.

Similarly, 5 LUAD patients were selected, 3 of which were NACT-SEN (3 CRs, 1 PRs) and 2 were NACT-NON-SEN (Fig. 7B).

In LUAD, BATF3 significantly down-regulated in the NACT-SEN group, while the expression of IRF5, ZBTB38 in the NACT-SEN group were significantly higher than the NACT-NON-SEN group in malignant epithelial cell cluster (marked with EPCAM and SOX4, Fig. 7D). In ESCC, BATF3, IRF5, ZBTB38 showed the same tendency in the 9801 malignant cells marked by LYZ and C1QB (Fig. 7C).

## Discussion

Cisplatin is one of the most potent and widely used drugs for the treatment of various solid cancers such as testicular, lung, cervical cancer, etc., [7], while tumor responses to cisplatin or carboplatin depend on the levels of platinum–DNA adducts and the DNA repair capacity of the cells [3, 15]. The development of cisplatin resistance in human cancer cells, including cell growth-promoting, apoptosis, DNA damage repair, and endocytosis, all of which are mechanisms supporting cell survival [16].

In this study, we began with exploring the important genes and miRNA of different cancer cell lines related to the IC50 value of cisplatin, based on which, we constructed the predicting model of the cisplatin resistance using the expressing value of 6 mRNAs, 3 of which, BATF3, IRF5, ZBTB38 were also verified in our own samples. All of them are closely related to the immune.

On the one hand, several studies reported that chemotherapy was able to modulate the function of TAM [17–19]. On the other hand, the different alteration of TAM reflects patients’ responses to chemotherapy [20–23]. Coincidentally, the three genes we selected are all related to immunity, which might suggest that they affect tumor cell sensitivity to cisplatin by regulating TAM. The interaction between BATFs and IRFs in immune cell lineages occurs in the gene expression network of several crucial processes. For example, BATF and IRF4 cooperate in CSR, as well as in antibody class switching through influencing T_FH_ cells and germinal center B cells [24]. What’s more, the cooperation of ZBTB46, BATF3, and IRF8 activates the development of CD8α+ conventional dendritic cells (cDCs) [25]. In this study, we found ZBTB38, BATF3, and IRF5 might have interactions that affect the cell sensitivity to cisplatin, but with various trends.

Basic leucine zipper transcription factor ATF-like (BATF), BATF2, and BATF3 belong to the activator protein 1 (AP-1) family of transcription factors, which regulate numerous cellular processes [26]. BATF3 was first identified in human T cells and was later found to play a critical role in the development of the cDC1 subset of conventional DCs [25, 27]. Although we found that knocking down the expression of BATF3 will increase cancer’s sensitivity to cisplatin, the main function of this transcription factor is to activate CD8alpha+ dendritic cells. In other words, it plays important role in cross-presentation in tumor rejection and deletion of the transcription factor Batf3 ablated development of CD8alpha + dendritic cells [28]. Furthermore, within the OpACIN trial, severe melanoma patients suffering from the reoccurrence of tumor after adjuvant or neoadjuvant consisting ipilimumab + nivolumab displayed a low level of Batf3^+^ DC-associated genes [29], which might reveal the two-side adjusting effects of BATF3 on chemotherapy and immunotherapy.

Interferon regulatory factor-5 (IRF5) is a transcription factor and has essential cellular mechanisms as a tumor suppressor gene [30]. The beneficial effects of NACRT on TAMs’ infiltration might be associated with gender-dependent IRF-5 expression, as CD163 + TAMs, which were related to poor prognosis[31], were shown to be negatively correlated with the number of IRF-5 + cells[20]. It was also reported that increased expression of IRF-5 in M2-like TAM promoted antitumor immune response to NACT [31]. In our research, IRF-5 showed the same tendency as the knocking down of it could increase tumor cell's resistance to cisplatin with a higher IC50, and the potential mechanism might lie on its function of secretin IFN-α, because the delivery of IRF5 protein into human primary pDCs increased IFN-α secretion [32], which has antiproliferative, differentiation-inducing, apoptotic, and antiangiogenic properties, and its clinical activity has been demonstrated in several cancers, including as post-chemotherapy maintenance [33, 34].

Zinc finger and BTB domain-containing 38 (ZBTB38) represents one member of the zinc finger (ZF) family of Methyl-CpG-binding proteins (MBPs) [35]. Similar to the IRF5, in our experiment, its expression is related to cisplatin sensitivity. Previous study showed that ZBTB38 can enhance the response to DNMT inhibitor therapies as a target of DNA methyltransferase inhibitor [36] as well as it can influence response of cancer cell lines to chemotherapy through involving in diverse epigenetic processes affecting DNA methylation [37]. However, The biological function of ZBTB38 remains also elusive [38]. In bladder cancer, ZBTB38 promotes migration and invasive growth [39], while in prostate cancer, depletion of ZBTB38 results in higher expression of ROS and elevated cell death after doxorubicin treatment [40].

However, the specific mechanism among those 3 genes, with which cancer cells' sensitivity to cisplatin could be changed, still needs to be explored.

miRNAs could modulate about 30% of gene expression through influencing mRNA translation [41], which play a key role in many biological processes, including tumor chemoresistance [42]. Evidence has shown that the expression of several miRNAs may relate to cisplatin resistance in malignant cells [43, 44]. In our study, miR-200c and miR-203 finally contributed to the cisplatin-resistance model. The former, which belongs to the miR-200 family, is reported that it could increase the sensitivity of cells to antitumor medications in a variety of cancers, including gastric [45, 46], breast [47], and non-small cell lung cancer [48]. Similarly, miR-203 was differentially expressed in DDP-sensitive and -insensitive tumor cells. Previous study has demonstrated that miR-203 could bind to the 3′UTR of DKK1 and then regulate the characteristics of lung cancer cells [49]. Furthermore, it also affects cisplatin resistance of pancreatic cancer cells [50], tongue squamous cancer [51]. In all, large numbers of surveys indicate that miRNAs actively affect the mechanism of cisplatin resistance.

## Conclusions

Somatic mutations, mRNA expressions, miRNA expressions, and metabolites differences were related to the resistance of cisplatin. The model we created and based on the expression of 3 genes, BATF3, IRF5, ZBTB38, could help in the prediction of the reaction and prognosis of cancer patients given platinum-based, especially cisplatin-including chemotherapies.

## Supplementary Information

**Additional file 1: Fig. S1.** Pan-cancer overview of cells' IC50 value of cisplatin.

**Additional file 2: Fig. S2.** The overall pattern of the mutation status.

**Additional file 3: Fig. S3.** (A) the model established by classification and regression tree (CART). (B) the model established by C4.5 decision tree classification algorithm.

**Additional file 4: Fig. S4.** The ROC plotters linking BATF3, IRF5, and ZBTB38 expression and response to therapy of breast and ovarian cancers.

**Additional file 5: Fig. S5.** Establishment of the LASSO model of miRNA.

**Additional file 6: Table S1.** Sequences of siRNAs targeting BATF3, IRF5, ZBTB38.

**Additional file 7: Table S2.** The sequences and melting temperature (Tm) of the primers used in our research, whether they span exon junctions, PCR efficiency and correlation with dilution series (R^2^).

## Data Availability

The data used to support the findings of this study are available either online or from the corresponding author upon request.
