# Supplementary figures and images for: Cisplatin resistance-related multi-omics differences and the establishment of machine learning models
Source: J Transl Med. 2022 Apr 11;20:171. doi: 10.1186/s12967-022-03372-0 (PMC9004122; doi:10.1186/s12967-022-03372-0)

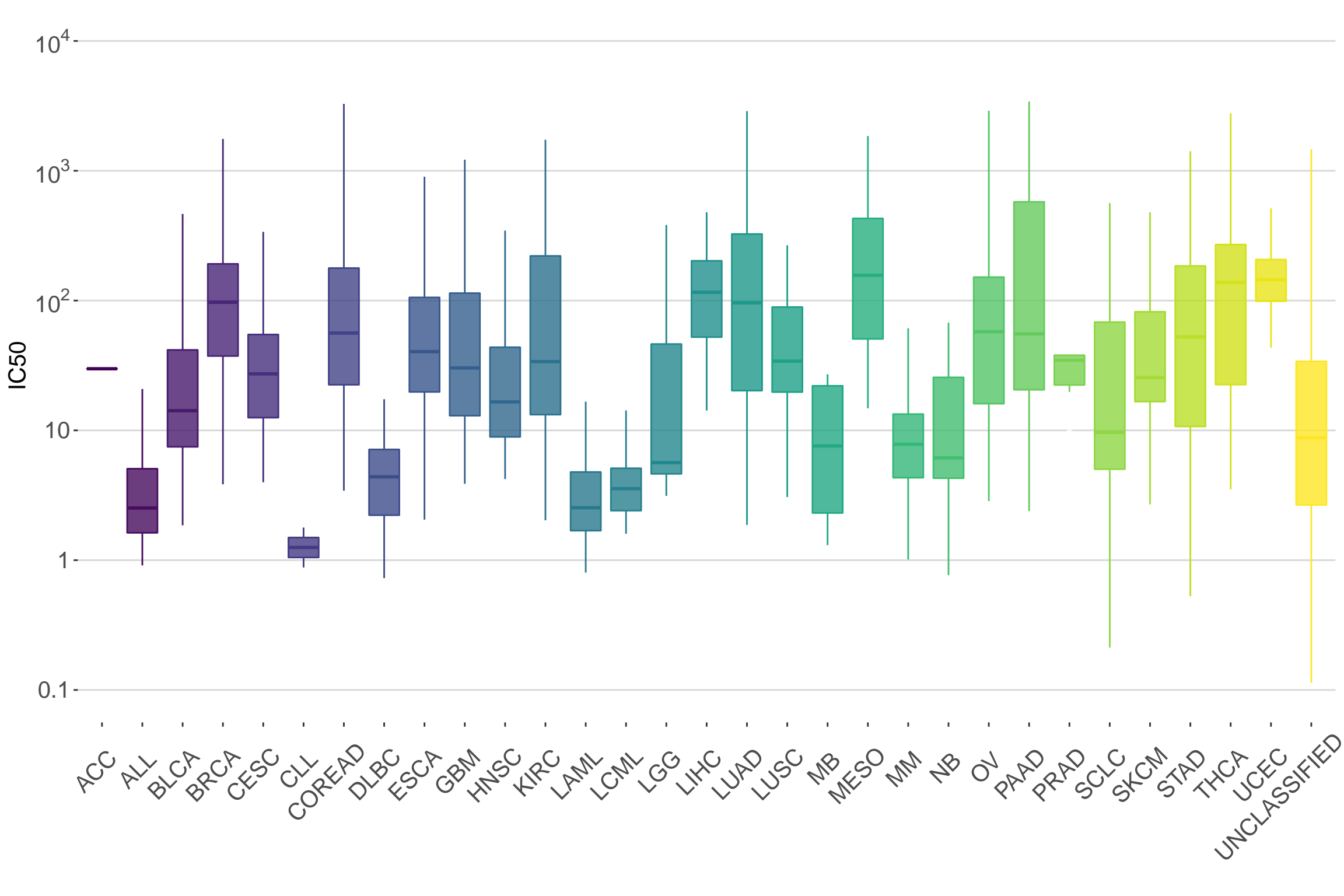

Supplement: Supplementary file 1 — Additional file 1: Fig. S1. Pan-cancer overview of cells' IC50 value of cisplatin. [file 12967_2022_3372_MOESM1_ESM.pdf]

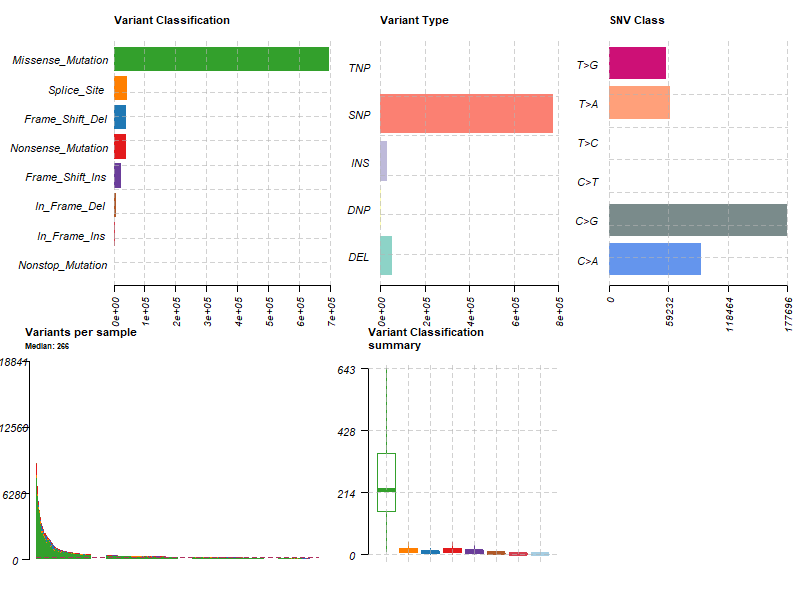

Supplement: Supplementary file 2 — Additional file 2: Fig. S2. The overall pattern of the mutation status. [file 12967_2022_3372_MOESM2_ESM.tiff]

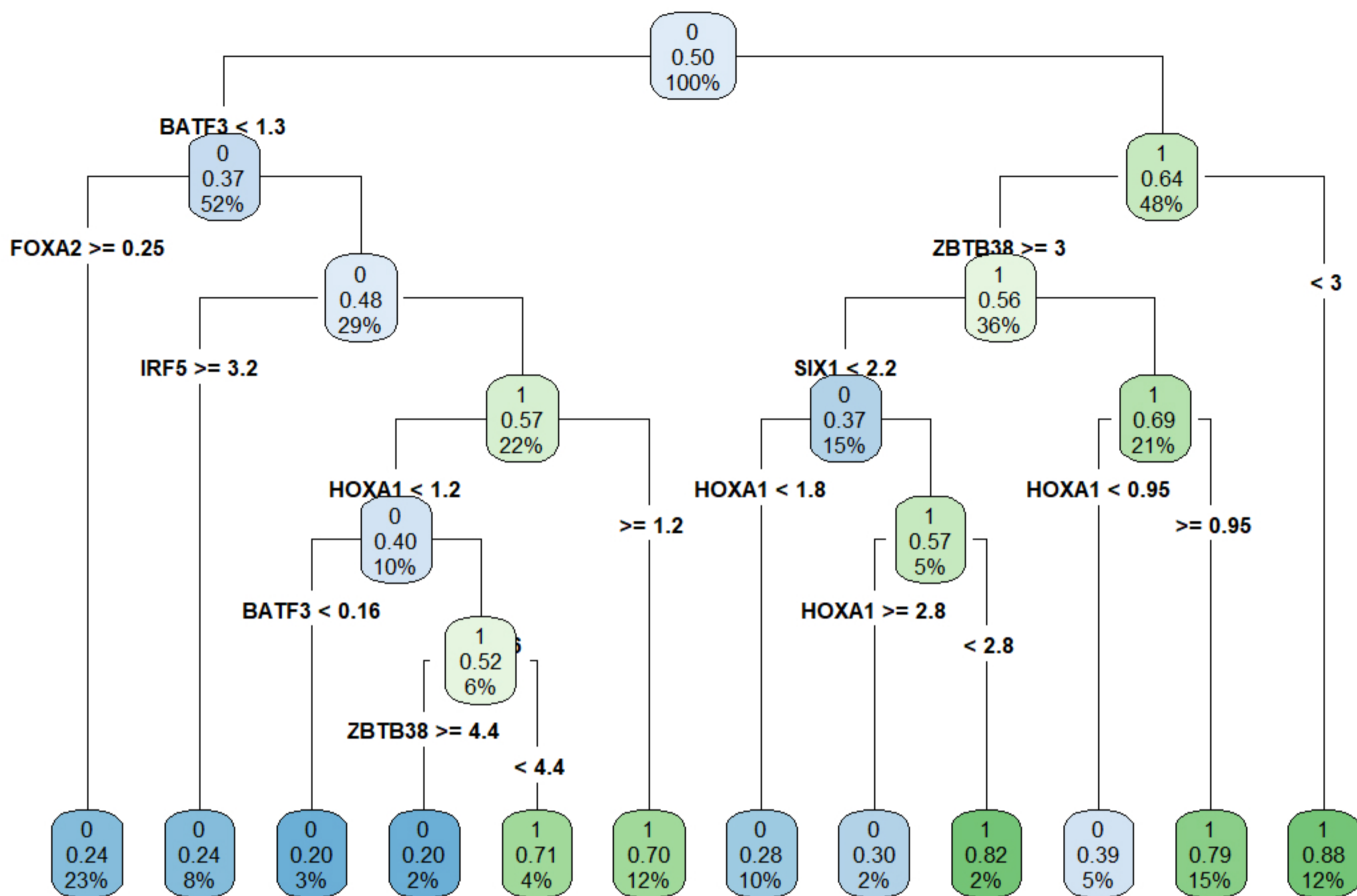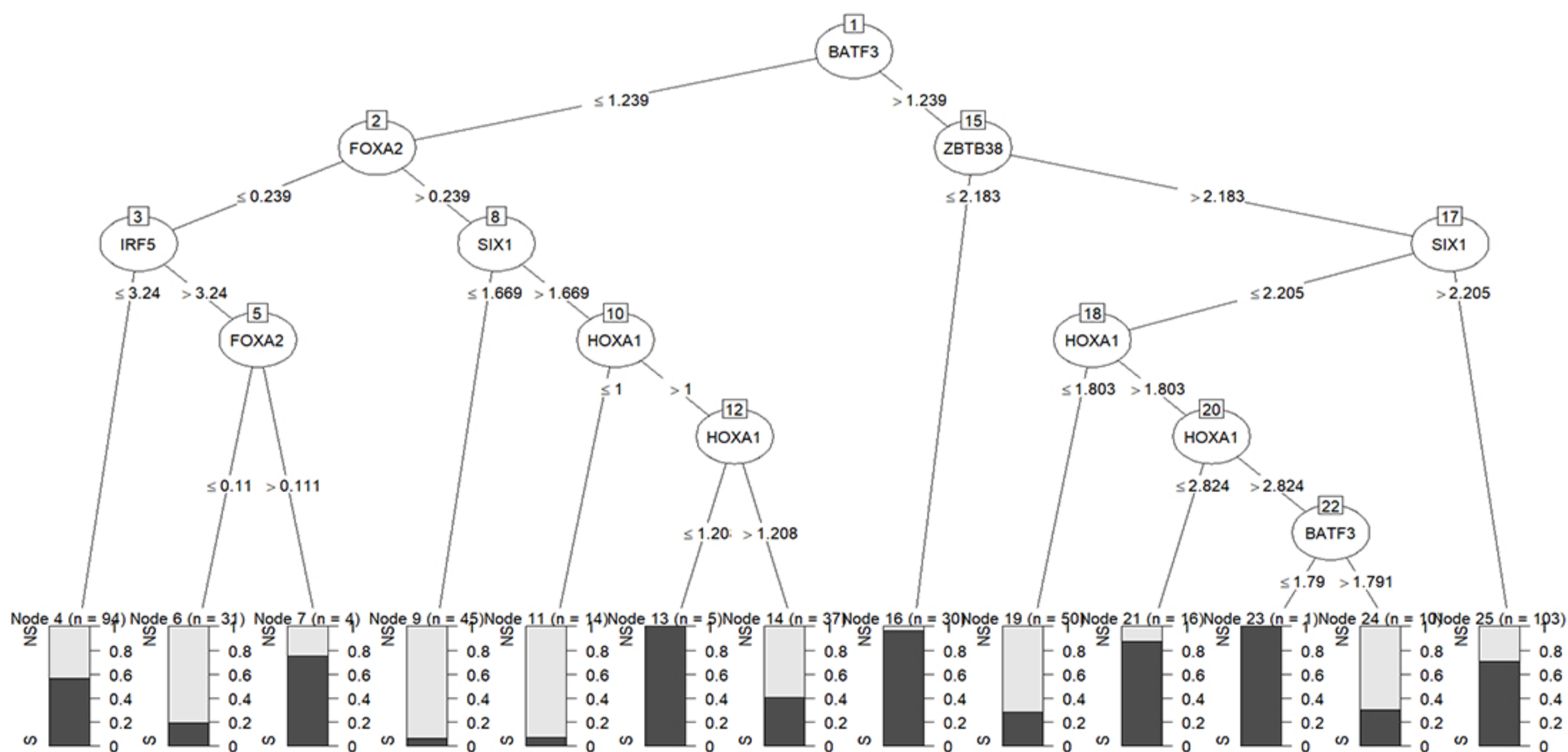

Supplement: Supplementary file 3 — Additional file 3: Fig. S3. (A) the model established by classification and regression tree (CART). (B) the model established by C4.5 decision tree classification algorithm. [file 12967_2022_3372_MOESM3_ESM.pdf]

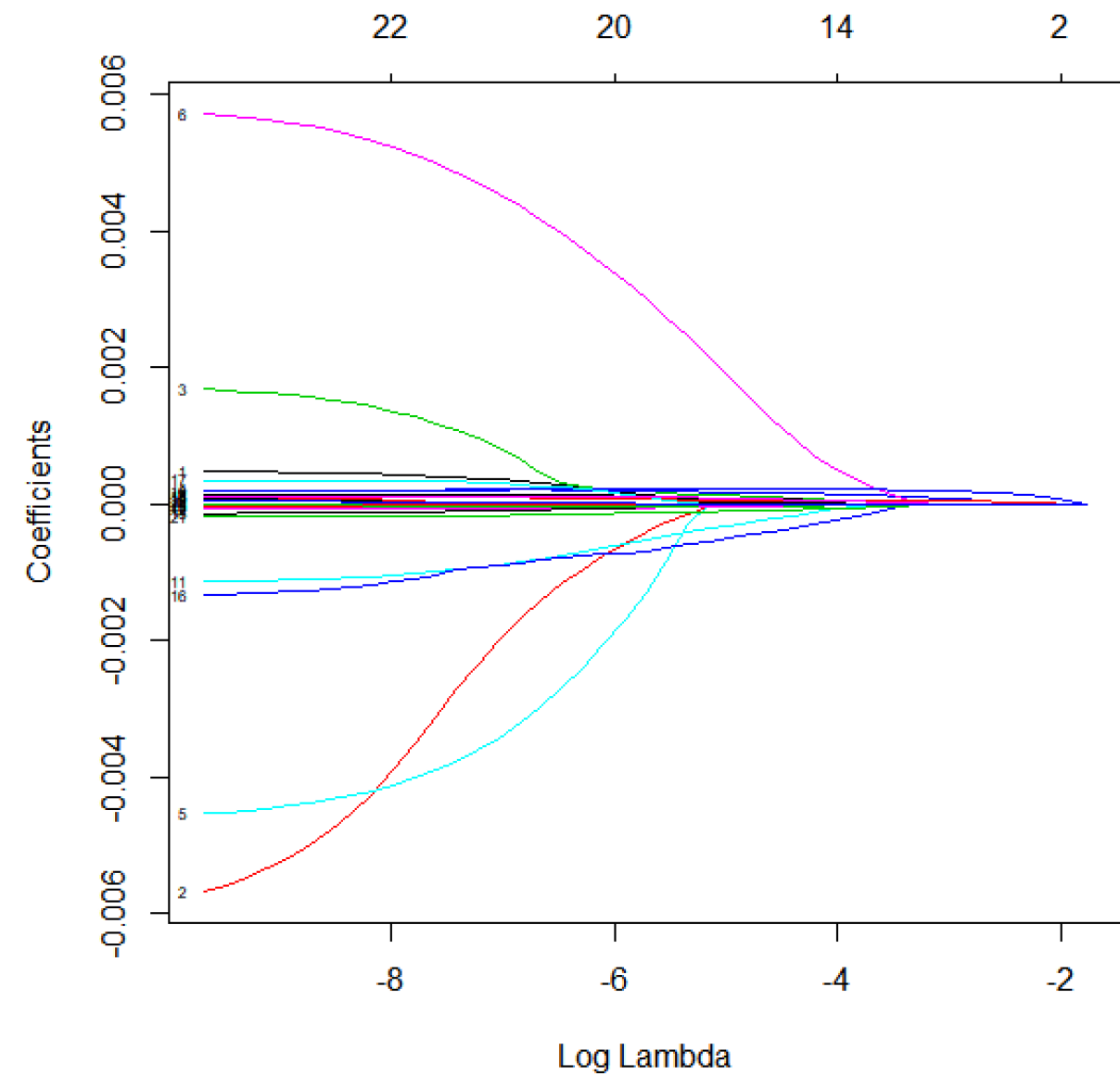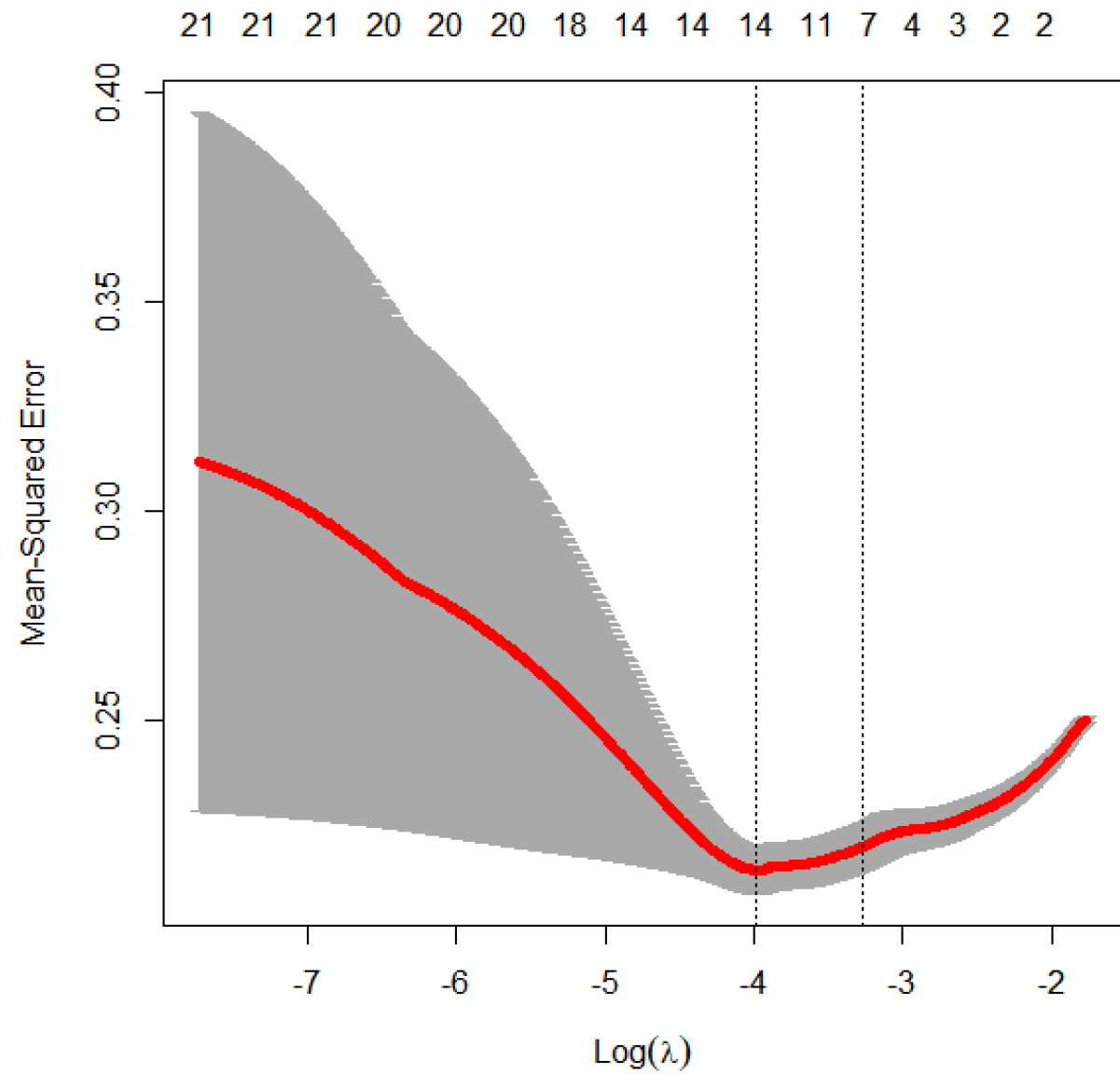

Supplement: Supplementary file 5 — Additional file 5: Fig. S5. Establishment of the LASSO model of miRNA. [file 12967_2022_3372_MOESM5_ESM.pdf]
