# Supplementary material for: Cisplatin resistance-related multi-omics differences and the establishment of machine learning models
Source: J Transl Med. 2022 Apr 11;20:171. doi: 10.1186/s12967-022-03372-0 (PMC9004122; doi:10.1186/s12967-022-03372-0)
Supplement: Supplementary file 6 — Additional file 6: Table S1. Sequences of siRNAs targeting BATF3, IRF5, ZBTB38. [file 12967_2022_3372_MOESM6_ESM.docx]

**Additional file 6: Table S1.** Sequences of siRNAs targeting BATF3, IRF5, ZBTB38

| Name | Targeting sequence |
| --- | --- |
| genOFFTM st-h-BATF3_001 | CGAGTTGCTGCTCAGAGAA |
| genOFFTM st-h-BATF3_002 | CTGCTCTGCCCTATGAACT |
| genOFFTM st-h-IRF5_001 | GGAAGTACTTTGCCAGACA |
| genOFFTM st-h-IRF5_002 | GAAGGCTCCTCAGGATAAA |
| genOFFTM st-h-ZBTB38_001 | CCTATTCCTTACCCAAAGA |
| genOFFTM st-h-ZBTB38_002 | GGGACAACACCATCTTCAA |
