# Supplementary material for: Cisplatin resistance-related multi-omics differences and the establishment of machine learning models
Source: J Transl Med. 2022 Apr 11;20:171. doi: 10.1186/s12967-022-03372-0 (PMC9004122; doi:10.1186/s12967-022-03372-0)
Supplement: Supplementary file 7 — Additional file 7: Table S2. The sequences and melting temperature (Tm) of the primers used in our research, whether they span exon junctions, PCR efficiency and correlation with dilution series (R2). [file 12967_2022_3372_MOESM7_ESM.docx]

**Additional file 7: Table S2. The sequences and melting temperature (Tm) of the primers used in our research, whether they span exon junctions, PCR efficiency and correlation with dilution series (R^2^)**

| Gene | Primers Sequence 5’-3’ (forward, reverse) | Tm (℃) | Exon junction span | PCR efficiency (%) | Correlation with dilution series (R^2^) |
| --- | --- | --- | --- | --- | --- |
| BATF3 | TGCTCAGAGAAGTCGGAAGAA | 58.8 | Yes | 100.8 | 0.999 |
|  | TGGCACAAAGTTCATAGGGCA | 60.8 |  |  |  |
| IRF5 | GGGCTTCAATGGGTCAACG | 59.6 | Yes | 99.4 | 0.992 |
|  | GCCTTCGGTGTATTTCCCTG | 60.2 |  |  |  |
| ZBTB38 | AGCGCATTCGGGGCATTTTA | 62.4 | Yes | 101.2 | 0.995 |
|  | GCTGCGAGATCAGTGACTGT | 59.2 |  |  |  |
